# A volcano reawakens after more than 100,000 years of “silent” magma reservoir growth

**DOI:** 10.1126/sciadv.aec9565

**Published:** 2026-04-22

**Authors:** Răzvan-Gabriel Popa, Olivier Bachmann, Marcel Guillong, Andrea Giuliani

**Affiliations:** ^1^ Department of Earth and Planetary Sciences, ETH Zürich, Zürich, Switzerland.; ^2^ Earth and Planets Laboratory, Carnegie Institution for Science, Washington, DC, USA.

## Abstract

Magmatic systems can remain dormant for tens of thousands of years, creating a misleading perception of extinction that complicates hazard forecasting. To identify drivers of protracted quiescence, we integrate geochemical, isotopic, and zircon geochronological data comprising over 1250 crystallization ages from 31 eruptions at Methana, an active volcano near Athens, Greece. This record allows us to link eruptive activity, magma reservoir evolution, and mantle source variations over 700,000 years. Here, extended repose correlates with increased metasomatism of the mantle wedge by slab-derived components. The longest quiescence at Methana (>100,000 years) coincides with substantial magma production that was preferentially trapped in the crust. We attribute this trapping to the generation of superhydrous melts (>6 wt % H_2_O) from a highly metasomatized mantle. These volatile-rich magmas undergo water saturation and crystallize during ascent, preventing eruption. Such trapping mechanisms can grow large magma reservoirs and may enable transitions from small stratovolcanoes to highly hazardous, caldera-forming systems.

## Teaser

Volcanoes can experience long periods of dormancy, of tens to hundreds of thousands of years, and wake up unexpectedly.

## INTRODUCTION

Volcanoes can undergo long periods of dormancy as they build up to their next eruptive events, often in the range of decades to thousands of years (*1*). If the pauses are longer than 10 thousand years (kyr), most polygenetic volcanoes or stratovolcanoes are considered extinct, the exception being the rhyolitic caldera-forming systems, which can remain dormant for tens of thousands of years (*2*). However, these temporal limits are relatively arbitrary as they are based on historical observations and on limited geochronological datasets, making them biased toward the most active systems (*2*). From this perspective, prolonged periods of quiescence can be deceptive, as humans have potentially settled around long-sleeping but still dangerous volcanoes.

As shown here, even typical volcanoes erupting magmas of intermediate composition, like those defining magmatic arcs all around our planet, can remain inactive for deceivingly long times (>100,000 years). This can be the case even when the mantle continues to melt and primary magmas are being recharged. This scenario would often lead to the classification of volcanoes as extinct, only to rekindle unexpectedly. Such a behavior is connected to the state of their subvolcanic magma reservoirs, which might require longer times to accumulate the volumes of magma needed for an eruption due to low injection rates (*3*). Alternatively, the reservoirs might saturate in gas and exsolve a substantial amount of volatiles that render them less reactive to recharge, hence lowering the eruption frequency (*4*–*7*). Last, subvolcanic systems might be approaching extinction, by cooling and slowly becoming less eruptible as the rate of deep magmatic recharge decreases to a level where the system cannot remain above the solidus (*3*, *8*–*11*). Therefore, relating magma chamber evolution to the phases of quiescence is essential to understanding the life cycle of volcanoes and to predict how the magnitudes and frequencies of future eruptions might change. This is particularly challenging, since magma chamber processes are largely obscured from direct observation.

An approach to this problem is to investigate past records of volcanic events. Chemical variations in the magmas, along with the spatial and temporal development of volcanic edifices can shed light on the different stages of magma reservoir evolution, including the factors controlling it (*12*). Studies that have explored this connection are generally focused on larger volcanoes, especially with caldera-forming events, biasing our knowledge toward the rarer magmatic systems which were able to develop large reservoirs with extensive rhyolitic melt- and gas-rich pockets (*13*–*23*). The stages of reservoir development involving magmas that are dominantly mafic to intermediate in composition, including nucleation and growth in the crust as well as the onset of volcanic activity, are poorly understood although most volcanoes on Earth are in this phase of evolution. Among these are the typical stratovolcanoes and dome-field volcanoes that erupt andesites and dacites in subduction zones (*24*).

The life cycles of these volcanoes contain a few critical stages that control their development and activity, including their evolution into the larger, more dangerous systems. Some key questions related to those stages are: What controls the nucleation of crustal magma chambers? What does a prolonged period of quiescence mean for the future activity of a volcano? Will magma chambers grow to sizes capable of feeding catastrophic explosive eruptions, or will they remain relatively small and trapped at high-crystallinity, near-death limbos? To explore these questions, we document the history of Methana volcano, an active system located in the Aegean Volcanic Arc, less than 60 km from the Greek capital city of Athens.

Methana has generated over 31 eruptions, 3 of which were explosive and 28 effusive, building a dome field over an area of roughly 8 km by 6 km. Owing to the architecture of the dome field, spatial overlap between distinct units is limited, as most domes are emplaced laterally rather than overlying older deposits. As a result, any sampling bias would mainly concern small-volume eruptions that could be locally buried, making Methana an excellent candidate for examining long-term volcanic eruption frequency and magma reservoir evolution.

**Fig. 1.**
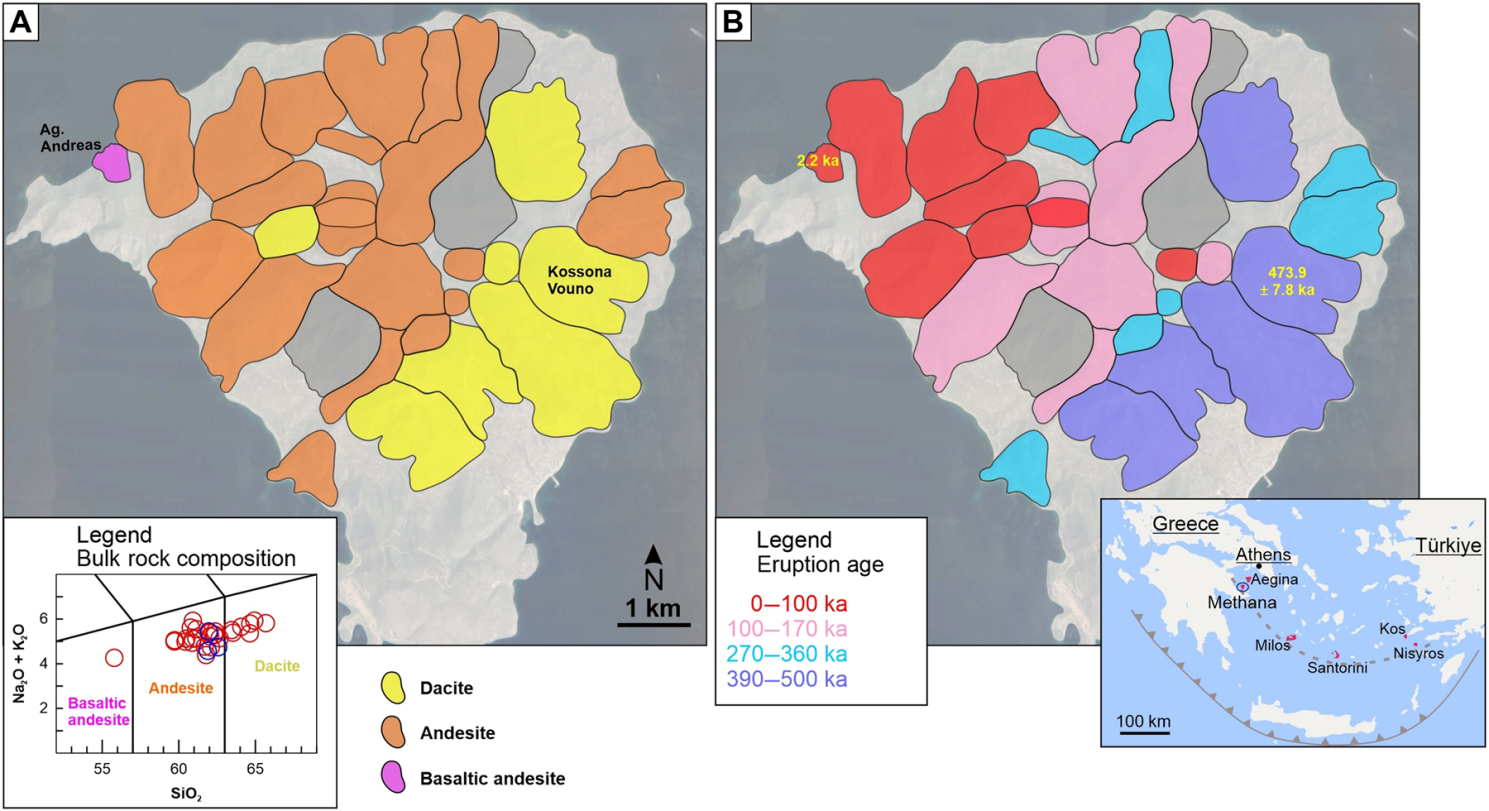
Overview of compositions and eruption ages of Methana. The compositional data (**A**) are based on the bulk rock x-ray fluorescence measurements shown in the total alkali versus silica (TAS) insert, where red circles depict effusive events, and blue circles are explosive eruptions. Yellow, orange, and purple colors indicate dacite, andesite, and basaltic andesite compositions, respectively. The eruption ages (**B**) are based on the youngest zircon populations. The pumice deposits are not shown on the map due to limited exposure. The oldest (Kossona Vouno) and youngest eruption (Agios Andreas) are indicated on the map (A), along with their ages (B). Two effusive eruptions, depicted in dark gray, have not been analyzed. The map inset (*25*) shows the South Aegean Volcanic Arc and the locations of the main volcanic areas.

The extruded products are generally intermediate in composition, ranging from andesite to dacite with rare basaltic andesite occurrences (Fig. 1). The effusive eruptions are generated from a high-crystallinity silicic upper-crustal reservoir, which requires sustained mafic recharges from the mid-to-lower crust to erupt (*25*). In contrast, the few andesitic explosive events and the rare basaltic andesite lava flows involve the direct eruption of mid-to-lower crust magmas that have experienced limited interaction with the subvolcanic reservoir (*25*). The timescale of volcanic activity at Methana is poorly known, except for the latest eruption witnessed 2250 years ago and described by the Greek historian Strabo (*26*), although its exact location is unclear.

To understand the evolution and growth of the magma reservoir and how this relates to its volcanic output and periods of quiescence, we rely on petrochronology. Using laser ablation inductively coupled plasma mass spectrometry (LA-ICP-MS), we apply well-established in situ U-Pb and U-Th disequilibrium dating methods on zircon crystals (*27*, *28*), recovering over 1250 crystallization ages that span a timeline of ~700 kyr. This allows us to characterize the crystallization history of Methana magmas and to approximate the eruption ages using the youngest zircon population in each unit (*6*). For the basaltic andesite, where zircon is absent, we rely on ilmenite U-Th disequilibrium dating (*29*). Geochronology is combined with bulk rock major and trace element compositions, along with in situ Hf isotopic data retrieved for 165 zircon and in situ Sr isotopic data on 525 plagioclase crystals, allowing us to investigate the polybaric connections between volcanic activity, subvolcanic reservoirs, and deep mantle sources.

## RESULTS

### Petrochronological overview

**Fig. 2.**
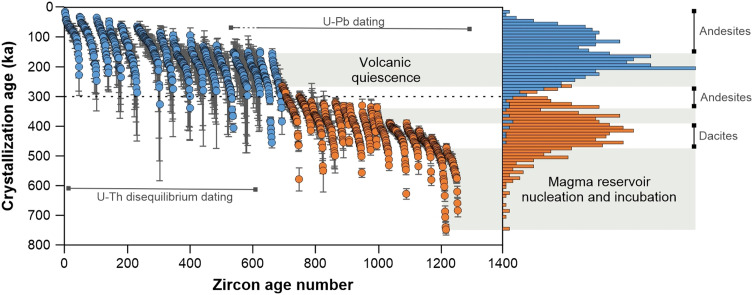
Snake plot depicting an overview of zircon crystallization ages obtained by in situ U-Pb and U-Th disequilibrium dating. The data points are grouped in near-vertical stacks (“snakes”) that represent different eruptions, the *x* axis showing the age rank order. The error bars are 2σ uncertainty. The histograms illustrate the main periods of zircon crystallization, which are interpreted as periods of intense intrusive activity. The minimum indicated by the histograms toward the present-day (0 to 50 ka) is accentuated by the undersampling of zircon due to limited eruptions. Orange and blue indicate the first and second eruptive cycles, respectively. Gray fields mark volcanic quiescence.

**Fig. 3.**
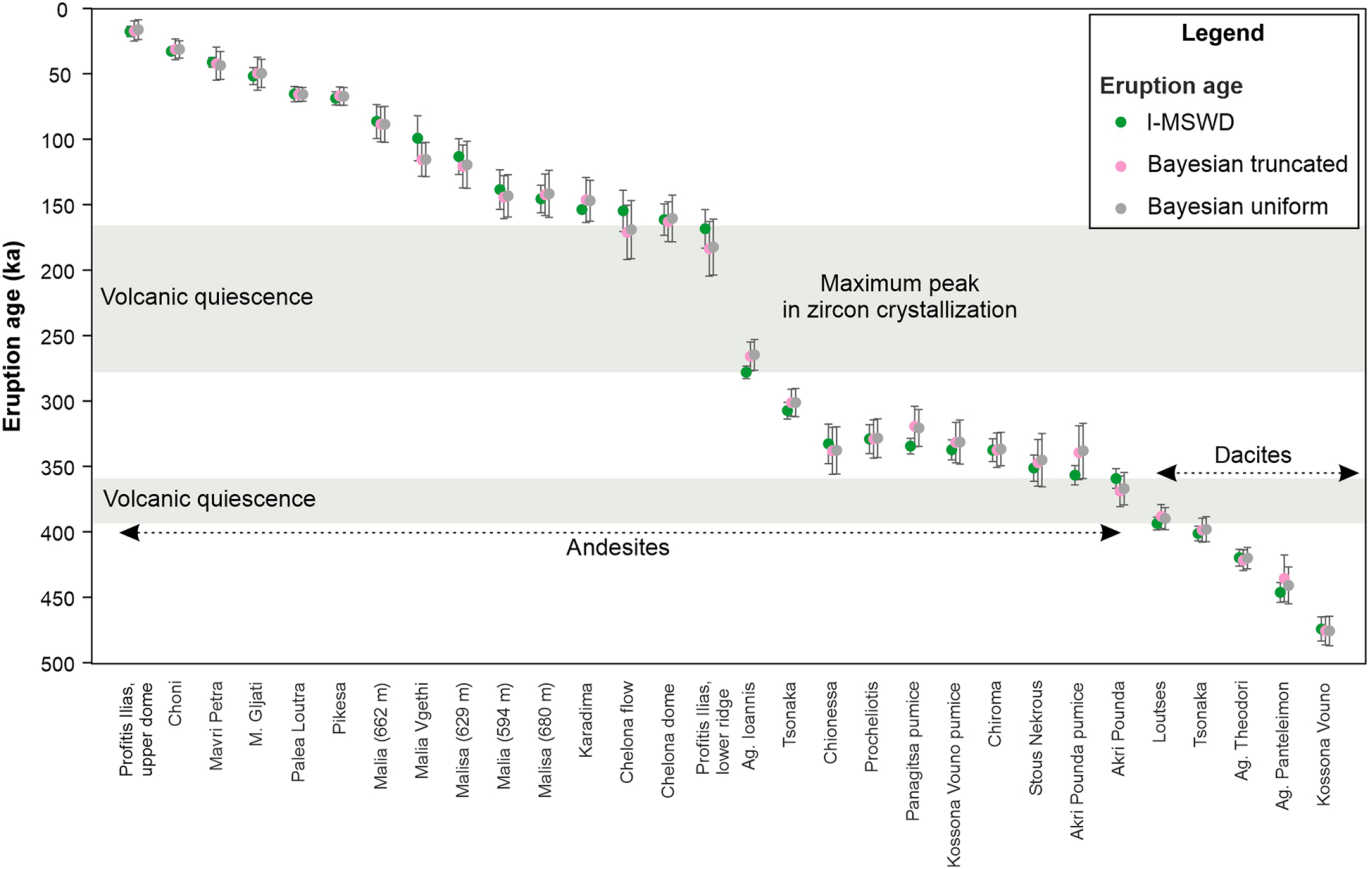
Overview of zircon-based eruption ages. The eruption ages are determined using the youngest population (I-MSWD) (*6*) and Bayesian methods (*30*). The methods agree, the ages being virtually indistinguishable from each other within 2σ uncertainty. The periods of volcanic quiescence are set based on the I-MSWD age. The local names of the volcanic units are provided for reference.

Our zircon data reveal an uninterrupted crystallization timeline (within the error of single grain analysis) of ~700 kyr, with no xenocrysts identified (Fig. 2). On the basis of the youngest population in each unit and on the distribution of model ages, using both the iterative mean square weighted deviation (I-MSWD) (*6*) and the Bayesian methods (*30*), we were able to estimate eruption ages, the two independent approaches showing good agreement (Fig. 3).

The zircon model ages indicate continuous crystallization for about 200 kyr before the first eruption that occurred on Methana, at 474 ± 8 thousand years ago (ka) (Figs. 2 and 3). After this initial dacitic extrusion, Methana experienced two main cycles of volcanic activity. The first cycle took place in the south-eastern and central part of the island and totaled at least 15 eruptions, including the explosive events. It began with scarce dacitic effusions, with five events recorded over a period of ~80 kyr. After a pause of ~30 kyr, between 393 ± 4 ka and 359 ± 7 ka, the activity ramped up and the frequency of eruptions effectively doubled. The compositions became slightly less evolved, with andesitic extrusions dominating until the activity ended at around 280 ka, marking the onset of a volcanic pause that lasted >100 kyr. This prolonged volcanic quiescence is the period for which we found the highest frequency of zircon crystallization (Fig. 2). Volcanic activity restarted at 168 ± 15 ka, beginning the second cycle, which was geographically shifted toward the central and north-western parts of the island. As opposed to the first cycle, volcanic activity was more intense in the early stages, with six eruptions taking place during a period of ~30 kyr, and waned toward the present-day, with 10 events spread over a period of ~140 kyr. The youngest eruption on the island is also the most primitive: a basaltic andesite that extruded in the north-western corner of Methana (Fig. 1). Lacking zircon, we dated this unit by the ilmenite U-Th method. Although this geochronological technique cannot precisely constrain such a young age [~2250 before present (BP)], the youngest isochron corresponds to the present-day and suggests that this is the historical eruption described by Strabo (see table S2).

**Fig. 4.**
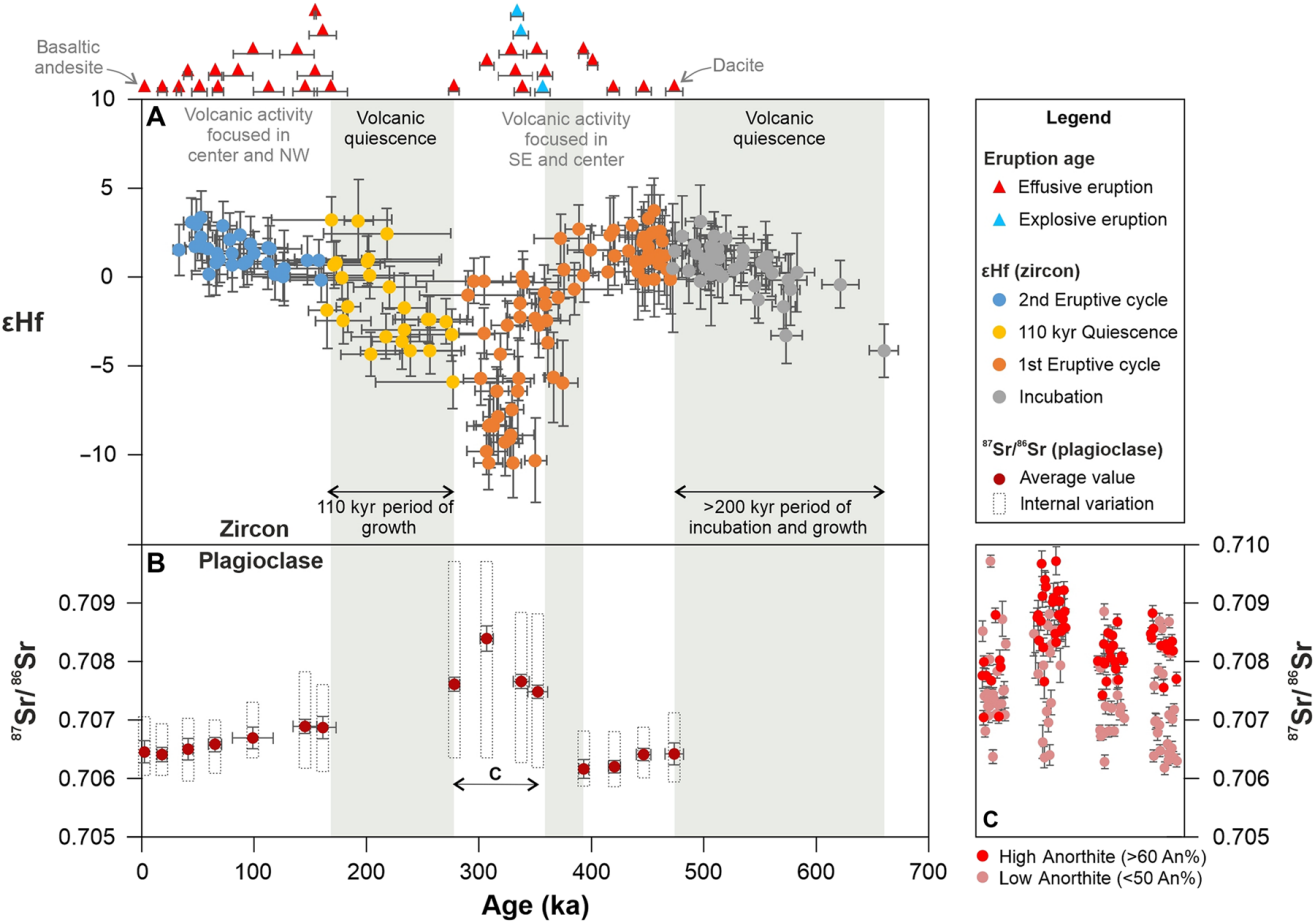
Zircon and plagioclase isotopic variability as a function of age and eruption frequency. (**A**) Hf isotopes of individual zircon grains expressed as εHf (i.e., per 10,000 variation from the chondrite uniform reservoir) (*31*). Error bars represent 2σ uncertainties. The red and blue triangles shown above the plot are marking the eruption ages (horizontal spread), while their vertical spread is simply for visualization purposes. (**B**) Average ^87^Sr/^86^Sr values of plagioclase (~40 analyses per selected unit), where error bars indicate SD of the mean of Sr isotope ratio (vertical) and eruption age (horizontal). The rectangles with dotted outline represent the total spread in ^87^Sr/^86^Sr. Although the plagioclase crystals sampled within the same eruption show some degree of Sr isotopic variability, potentially related to the protracted crystallization of this mineral, there is no clear distinction between rims and cores. (**C**) In the units showing high ^87^Sr/^86^Sr isotopic variability [age range defined as “c” in (B)], the high anorthite recharge crystals are generally limited to highly radiogenic compositions (>0.708), while the low anorthite plagioclase span the entire range of isotopic variability. NW, northwest; SE, southeast.

The magmatic stages identified at Methana, including the two eruptive cycles and the main periods of quiescence, correlate with the Hf isotopic record of zircon (Fig. 4A), which is expressed as εHf (*31*). Over the initial ~200 kyr period of noneruptive crustal magmatic activity, εHf increases steadily from values of approximately −4 to +2, trending toward more “mantle-like” (i.e., chondritic to marginally suprachondritic) values. Then, during the first eruptive cycle, after the 30 kyr of quiescence, εHf decreases sharply over a timescale of 100 kyr, reaching “crustal-like” values as low as −10 and showing significant variability within the same volcanic unit (up to values of around 0). This decrease in εHf marks the transition from dacitic to slightly more primitive, andesitic eruptions.

The ^87^Sr/^86^Sr isotopic data recovered from plagioclase crystals (Fig. 4B) show a clear anticorrelation with zircon εHf. Positive εHf anomalies correspond to the lowest Sr isotopic ratios (average ^87^Sr/^86^Sr between ~0.7062 and ~0.7064 for different units; *n* = 11), and negative εHf corresponds to the most radiogenic Sr signatures (average ^87^Sr/^86^Sr between ~0.7075 and ~0.7084; *n* = 4). While most units show limited ^87^Sr/^86^Sr variability between individual crystals (<0.001), the eruptions recording higher average radiogenic Sr also have a larger internal spread (up to 0.003), mirroring the higher internal spread observed in the zircon εHf data for the same units. The high-Anorthite plagioclase (An% > 60), which represent recharge crystals originating from the mid-to-lower crustal reservoirs (*25*), show lesser Sr isotopic variations than the low-Anorthite phenocrysts (An% < 50) and are limited to more radiogenic compositions (Fig. 4C).

Following the period when the Hf and Sr isotopes reached their most crustal-like values, the 110 kyr of quiescence ensued, and the highest frequency of zircon crystallization took place. During the volcanic quiescence, Hf and Sr isotopes progressively evolved back toward less enriched values (i.e., more mantle-like). The beginning of the second eruptive cycle was marked by a change in the slope of the εHf and ^87^Sr/^86^Sr trends, which remained approximately flat over the next ~170 kyr, implying more steady mantle conditions associated with the recent eruptive cycle.

**Fig. 5.**
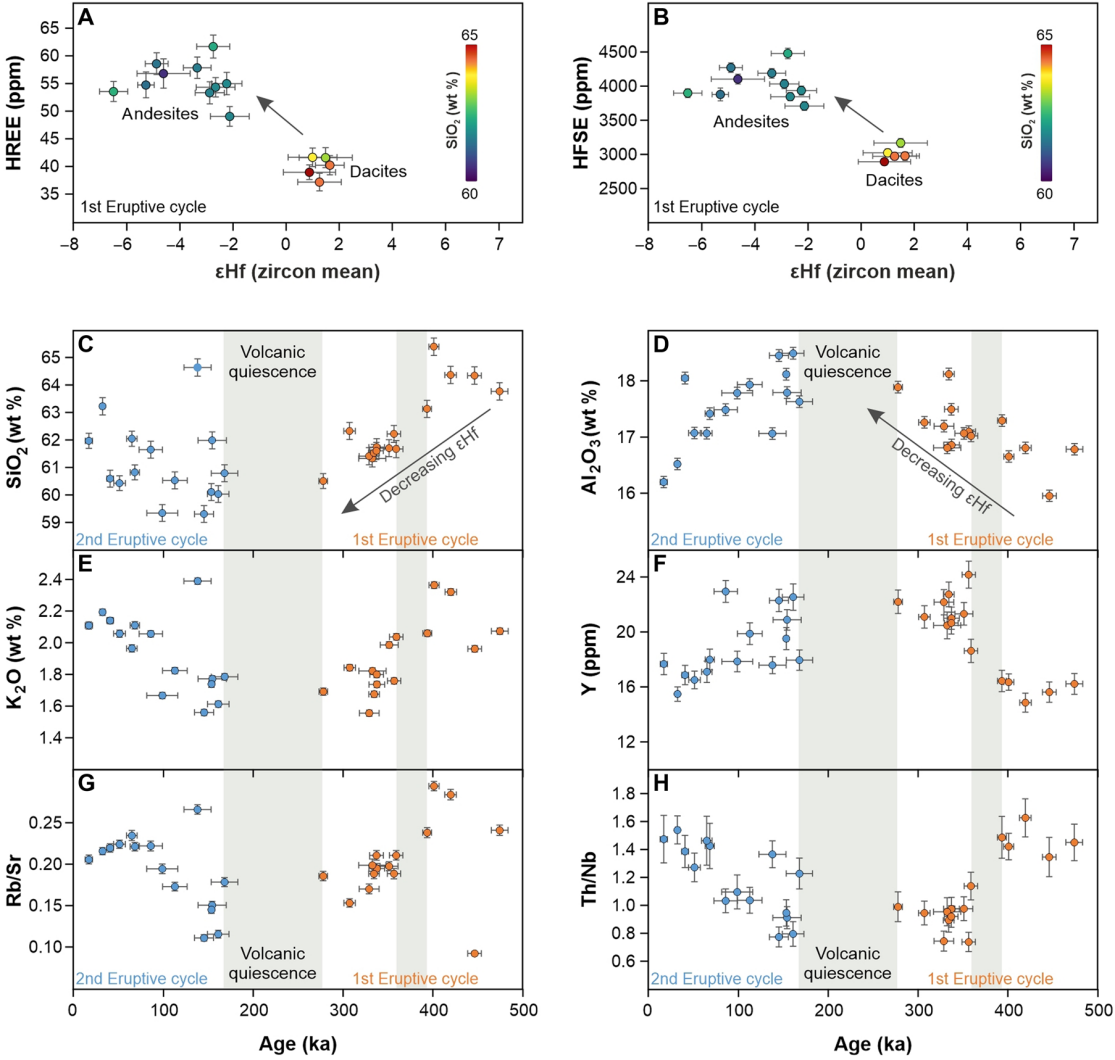
Geochemical evolution of the Methana volcanic system. Bulk rock geochemical data are compared to mean εHf values of zircon (**A** and **B**) and to eruption ages (**C** to **H**). The εHf value for each eruption is the weighted mean of the individual zircon values that correspond to each eruption age, within uncertainty. The HREE is the sum of Eu-to-Lu, including Sc and Y which have the same geochemical behavior, while the HFSE is the sum of Ti, Zr, Hf, Nb, and Ta. In (A) and (B), the symbols are color-coded based on bulk rock SiO_2_ content. In (C) to (H), orange and blue colors stand for first and second eruptive cycle, respectively, while gray fields indicate the periods of volcanic quiescence. Error bars represent 2σ uncertainties. ppm, parts per million.

Additional insights are provided by correlations of these isotopic variations with bulk rock major oxide and trace element concentrations. The isotopic “crustal” signature marked by decreasing zircon εHf and increasing plagioclase ^87^Sr/^86^Sr correlate with a 50% increase in the concentration of heavy rare earth elements (HREE) and high field strength elements (HFSE) in the bulk rocks (Fig. 5, A and B), while the HREE/MREE ratios remain mostly unchanged. This trend is coupled with increasing concentrations of elements that are generally compatible in magmas of intermediate composition (e.g., Al_2_O_3_, and Y) and with decreasing SiO_2_ and K_2_O, indicating a shift to more primitive bulk rock compositions (Fig. 5, C to F). The isotopic crustal signature also corresponds to a noticeable decrease of Rb/Sr and Th/Nb (Fig. 5, G and H).

## DISCUSSION

### Three critical observations on the importance of magma storage and mantle recharge at Methana

A first notable observation is that the earliest volcanic activity at Methana lacked mafic eruptions and was preceded by a prolonged, ~200 kyr period of zircon crystallization. On the one hand, this indicates that the primitive magmas produced in the mantle did not make it to the surface. On the other hand, it shows that Methana underwent a stage of magma reservoir nucleation and initial growth in the crust, without identifiable extrusive activity, which allowed the differentiation of these primitive magmas to more silicic, dacitic compositions. The incubation and maturation period may have lasted even longer than 200 kyr, as zircon is not stable in the most hot and mafic, mantle-derived magmas (*32*). As a result, our older crystallization record might be incomplete. Nevertheless, mineral chemistry in the early-erupted dacitic units indicates that this initial incubation period led to the formation of a complex plumbing system, including a subvolcanic silicic mush, which, based on amphibole-plagioclase thermobarometry, was stored at pressures of ~2 kbar, with magma chamber storage temperatures ranging between ~780° and 820°C (*25*). This high crystallinity magma was relatively immobile, and on the basis of hybrid postmixing mineral compositions and on the presence of mafic enclaves, it required at least two subsequent pulses of primitive recharge (basaltic andesite, ~1000°C) to be mobilized and generate the first eruptions (*25*).

The second notable observation concerns the relationship between compositional variations and eruptive behavior. Periods of frequent eruptions and of quiescence correlate with shifts in magma chemistry, suggesting that volcanic activity was modulated by processes that might have changed the chemical and isotopic signature of the magmas. Specifically, as zircon and plagioclase isotopic signatures trended toward more crustal-like values, eruptive activity initially intensified but eventually paused altogether when the most crustal signatures (i.e., lowest εHf and highest ^87^Sr/^86^Sr) were recorded. In the Aegean Arc as a whole (*33*–*35*), and at Methana in particular (*36*), it has been shown that the isotopic signatures are mostly controlled by addition of subducted crustal material to the mantle source, as opposed to path contamination during crustal transit (*37*, *38*). In the Aegean Arc, this process is likely enhanced by the exceptionally thick sedimentary cover on the subducting plate (up to 8 km at the Hellenic Trench) and by the highest sediment subduction rate documented globally (*36*, *39*). Changes in the composition of the mantle source during the lifetime of a volcano have also been observed in other volcanic areas, such as the Andes, at Tungurahua (*40*) and Pichincha (*41*, *42*).

Our data support this interpretation, with plagioclase serving as a first-order discriminant of the locus of crustal input. At Methana, the most radiogenic ^87^Sr/^86^Sr ratios are recorded by recharge crystals originating from the mid-to-lower crustal levels of the magmatic system (Fig. 4C), indicating that significant contamination did not occur in the upper crust but originated from the deeper parts of the system. Bulk rock geochemistry provides additional constraints, as the increase in HFSE concentrations which parallel the development of crustal-like isotopic signatures (Fig. 5B) is more readily related to mantle processes than crustal contamination at shallower levels. This is consistent with the shift to more primitive compositions recorded by major oxides and trace elements, as the lower εHf isotopic values are observed in andesites, not dacites. Crustal contamination from silicic rocks in the upper plate would increase both SiO_2_ and K_2_O, as well as Rb/Sr and Th/Nb, which is at odds with our observations. Hence, the notable decrease in εHf and increase in ^87^Sr/^86^Sr suggest melting of a part of the mantle that was more strongly metasomatized by sediment-derived fluids and melts, which would also explain the increase in HREE concentrations (Fig. 5A). Percent amounts of slab sediment melting would be probably sufficient to influence the Hf isotopes of Methana lavas, as shown elsewhere (*43*, *44*).

The third key observation is that the interval of peak zircon crystallization coincides with Methana’s longest eruptive quiescence of ~110 kyr. Such a pause could reflect a dearth of mantle melting and limited supply of recharge magma to sustain the upper-crustal reservoir and the triggering of eruptions. However, this is at odds with the zircon bloom observed during the same period. A peak in zircon crystallization is expected to occur when the reservoir reaches the optimum temperature and melt composition for zircon growth. This optimal temperature lies just below the zircon saturation temperature (*45*), approximately between 800° and 880°C (*46*), which is relatively warm for upper-crustal storage and typically requires ongoing recharge to be maintained. On the other hand, if magma production in the mantle would decrease substantially, then it would lead to a reduction of recharge to the upper crust and the system would cool progressively toward the solidus, below the optimal range for zircon growth. Although zircon can continue to crystallize, the lower temperatures, reduced diffusion rates, and depletion of zirconium from the melt would not favor the development of a pronounced crystallization peak. For this reason, we consider a simple waning of mantle melt supply less consistent with the data than a scenario involving sustained recharge during the eruptive hiatus. The sustained recharge hypothesis is further supported by the close association in time between the period of quiescence and zircon bloom and the most crustal-like isotopic signatures, which we attribute to the melting of a highly metasomatized mantle domain. Given that everything else remains equal (pressure-temperature conditions), this scenario would enhance magma production rates and recharge fluxes and explain the zircon bloom observed during the period of volcanic pause. Therefore, at Methana, a higher recharge rate appears to correlate with the period of extended quiescence, a paradox explored below.

### Superhydrous melts and the “silent” nucleation of magma reservoirs

When magmatism is initiated, mantle-produced mafic magmas are expected to migrate into the lower crust, where they can form early storage regions (*47*). From there, they eventually ascend through a thermally immature upper crust, which initially lacks the ability to host magma bodies of substantial volume (*21*, *48*). This is expected to result in frequent eruptions of mafic magmas, accompanied by the formation of short-lived intrusions of primitive compositions with high cooling and evacuation rates (*21*). Over hundreds of thousands of years, such mafic intrusions decrease the viscosity of the host rocks by heating them, leading to the thermal maturation of the middle to upper crust (*9*, *49*), which, in turn, renders the intrinsically brittle rocks more prone to accommodating the sequential growth of a magmatic reservoir (*3*, *5*, *50*, *51*). Such a progression, inferred from the early eruption of mafic magmas, is observed at several volcanic provinces around the globe but mostly in relatively dry tectonic settings, such as rift and intraplate zones (*52*). However, in continental arcs, where the mantle is fluxed by fluids or melts derived from the subducted plate, this initial stage of mafic volcanism tends to be rare or absent when water-rich, calc-alkaline compositions are involved (*53*). Magmatic provinces containing wet calc-alkaline magmas typically start with relatively differentiated intermediate to silicic (andesite/dacite) magmas, as exemplified by the Aegean Arc in Greece (*33*), by the San Juan Volcanic Field in the United States (*54*) or by the postcollisional Neogene volcanism of the Apuseni Mountains in Romania (*55*). This indicates that there should be a mechanism leading to the trapping of mantle-derived H_2_O-rich magmas in thermally immature crusts, common to many arcs.

Methana is notable in this regard, as our data indicate a “silent” nucleation of its subvolcanic reservoir, the initial incubation not being associated with any identifiable eruptive record. Here, the first products to extrude indicate the presence of an intermediate to silicic upper-crustal reservoir storing relatively evolved dacitic magmas, while the most primitive material (basaltic andesites) erupts exclusively in the youngest periods of activity. The key to explaining this conundrum could lie in the presence of high-Mg, high-Al amphibole in the recharge magmas of Methana.

**Fig. 6.**
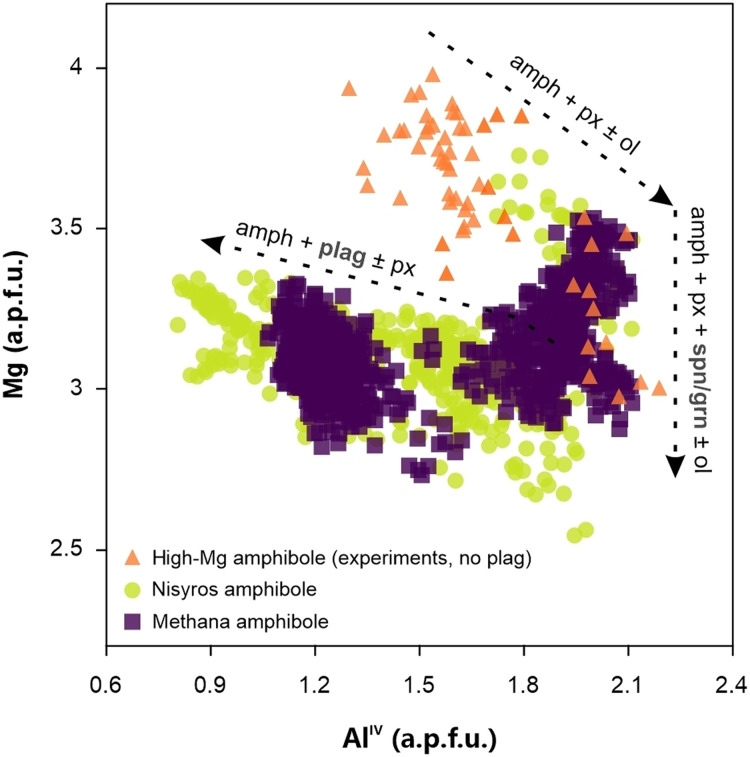
Comparison of natural and experimental amphibole compositions. The figure contains a compilation of electron microprobe data from Methana (*25*) and Nisyros (*102*), expressed as atom per formula unit (a.p.f.u.), compared to amphibole from experimental studies performed under superhydrous conditions (~1000°C, 3 to 10 kbar and 5.5 to 10 wt % dissolved H_2_O) (*56*–*59*). The Methana amphiboles that overlap with experimental high-Mg compositions are found exclusively in effusive units. Although they are not present in every dome, they appear in effusive products from both eruptive cycles. amph, amphibole; px, pyroxene; ol, olivine; spn, spinel; grn, garnet; plag, plagioclase.

Methana hosts amphibole populations that record distinct magmatic environments and crystallization conditions (Fig. 6). On the basis of previous thermobarometric estimates (*25*), the crystals that formed at relatively cold and stable subvolcanic conditions usually contain low Al^IV^ [1 to 1.5 atom per formula unit (a.p.f.u.)] and variable Mg (2.7 to 3.4 a.p.f.u.), while the hybrid crystals formed as a result of recharge events contain higher Al^IV^ of 1.6 to 1.9 (a.p.f.u.), at a similar range of Mg. Recharge amphibole typically exhibits the highest concentration of both elements, with 1.9 to 2.1 Al^IV^ and 2.9 to 3.5 Mg (a.p.f.u.). Among them, the crystals with the most elevated concentrations of Al^IV^ of around 2 to 2.1 (a.p.f.u.) show a steep variation over the entire range of Mg. According to experimental work (*56*–*59*), this chemical behavior is characteristic of near-liquidus mafic amphibole that crystallizes together with pyroxene, olivine, and a spinel or garnet phase, driving the variations in Mg, while the scarcity or absence of plagioclase buffers the Al content of the melt to elevated values. This scenario is specific to the stabilization of amphibole in hot (>950°C) mafic magmas, at mid-to-lower crustal pressures and under superhydrous melt conditions (*56*, *58*, *60*–*62*). These melts can hold >6 to 8 wt % H_2_O (*56*, *62*, *63*), which will be dissolved at high pressure (6 to 7 kbar) but will exsolve upon ascent toward the upper crust (*64*). The induced water exsolution makes superhydrous melts particularly difficult to identify, as they constantly reequilibrate their dissolved water content to shallower conditions (*65*) and remain elusive in the absence of mineral discriminants like amphibole (*66*). Despite this, they might play an important role in explaining our observations regarding the silent nucleation of the subvolcanic magma reservoir of Methana.

**Fig. 7.**
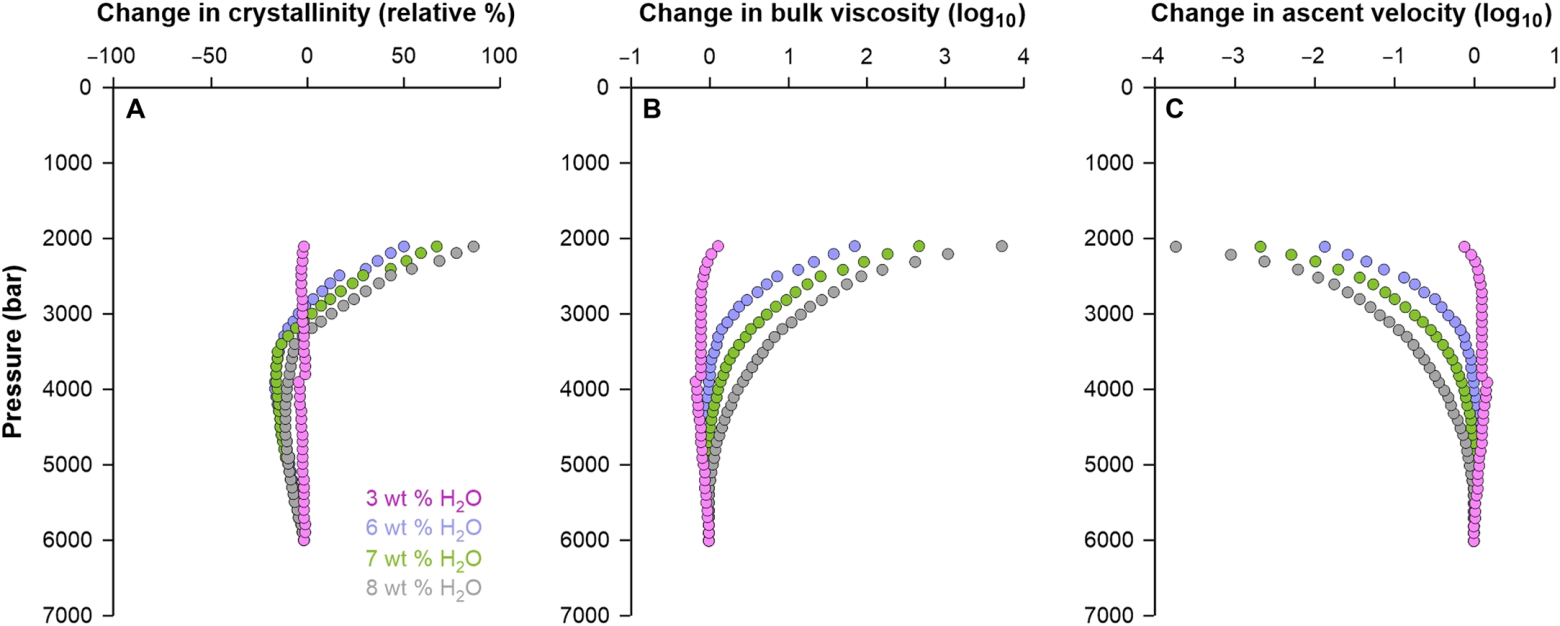
Changes in the physical parameters of magmas as a function of initial dissolved water content, during ascent from the mid-to-lower crust (6 kbar) to the upper crust (2 kbar). Changes in crystallinity (**A**) and bulk viscosity (**B**) are modeled using rhyolite-Melts (*70*, *71*), and the ascent velocity (**C**) is calculated using equation 4.45 of (*69*).

H_2_O saturation during decompression affects crystallization, melt and bulk magma viscosity, and ascent velocity (*63*, *67*–*69*). To simulate the physical evolution of magmas beneath Methana, we follow a recent example (*63*) and assess the role of H_2_O by using rhyolite-Melts (*70*, *71*). We run isothermal decompression simulations of basaltic andesites at 950°C, from an initial storage pressure of 6 kbar to a subvolcanic pressure of 2 kbar. Melt composition and initial storage conditions are based on the rare basaltic andesite occurrences on Methana (*25*). We performed four simulations at different initial dissolved H_2_O contents of 3, 6, 7 and 8 wt %, each containing 500 parts per million of CO_2_. For the H_2_O-poorest starting composition, the ascent is accompanied by equilibrium crystallization at each step of decompression (100 bar), which is counteracted by decompression-induced resorption of previously formed crystals, overall maintaining the crystallinity and, consequently, viscosity and ascent velocity quasi-constant (Fig. 7). On the other hand, the superhydrous magmas (H_2_O ≥ 6 wt %) saturate in water at between 5 and 4 kbar, which leads to crystallinity increasing shortly after H_2_O exsolution (Fig. 7A). These magmas double their crystal content by the time they reach 2 kbar (from 15 to 20 vol % crystals at 6 kbar to >30 vol % crystals at 2 kbar). Higher crystallinity increases bulk viscosity by three orders of magnitude (Fig. 7B) and consequently decreases the ascent velocity of the magmas by a factor of 100 to 1000 (Fig. 7C). The presence of >30 vol % crystals creates a physical framework that allows efficient gas permeability, thus enhancing the decoupling between exsolved gas and magma (*72*, *73*). This process of gas removal counteracts the acceleration potential associated with gas exsolution and buoyancy increase. The positive feedback between H_2_O exsolution, degassing via decompression, undercooling-driven crystallization, and increase in bulk viscosity acts to slow down the magma until it effectively stalls in the upper crust, in agreement with previous hypotheses (*62*, *63*, *74*, *75*). Such a process can aid the nucleation of a magmatic reservoir in a relatively cold upper crust that is not yet thermally matured.

In continental arcs, the formation of superhydrous mafic melts occurring in the initial stages of melting of a metasomatized mantle source could commonly result in the nucleation of magma reservoirs with limited to negligible concurrent eruptive activity. While decreasing the initial volcanic-plutonic ratio down to virtually 0, this process provides a mechanism for the silent appearance of magma reservoirs in the crust and explains the lack or scarcity of mafic magmas in the early stages of subduction-related magmatic provinces. Intermediate to silicic magmas (andesites or dacites) occur instead, following a stage of differentiation in the crust.

### The meaning of prolonged volcanic quiescence: Growth, death, or rejuvenation

The hypothesis described above can help answer another key question, namely, the occurrence of extended periods of volcanic quiescence such as the 110 kyr pause recorded at Methana. This noneruptive period correlates with the main peak of zircon crystallization (Fig. 2), which, as discussed above, indicates not only that the magmatic system was active but also that it was at its prime, being characterized by extensive magma trapping in the crust. The onset of this period closely follows the maximum amount of mantle source contamination via subduction, as recorded in this volcanic area by zircon Hf and plagioclase Sr isotopes (Fig. 4). We infer that this period is also correlated with intense hydration of the mantle wedge, as would be expected. Hence, after the initially scarce volcanic activity between 474 ± 8 ka and 393 ± 4 ka, marked by a relatively depleted mantle signature, the source shifts toward progressively more metasomatized mantle compositions over the following 100 kyr, due to increased subducted input. At this time, the volcanic-plutonic ratio increases, and all the explosive events on Methana occur, triggered by deep, relatively “damp” magmas, which we generally define as having intermediate dissolved water contents of 4 to 5 wt % (*7*). On the basis of previous hygrometry estimates (*25*), this change in eruption frequency suggests a shift from the low-volume production of less hydrous magmas (~3.5 wt % dissolved H_2_O) to a higher flux of slightly wetter, damp recharge magmas (4 to 4.5 wt % H_2_O), which is consistent with a more hydrated mantle source.

**Fig. 8.**
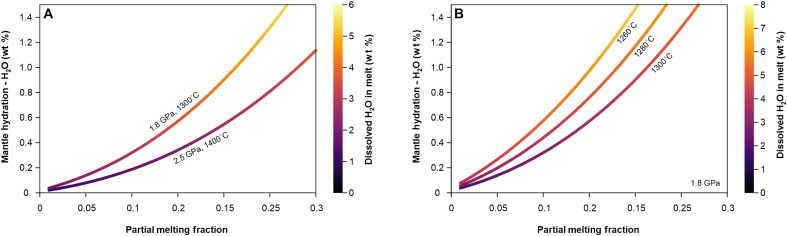
Pressure-temperature-H_2_O–dependent partial melting models of the mantle. We follow (*77*) and use the input parameters for a previously depleted mantle scenario that is subsequently hydrated. The color scale indicates the dissolved water content of the resulting primary melts, considering a partition coefficient of 0.01 for water. The P-T ranges explored in plot (**A**) are characteristic of what is expected in the Aegean Arc, with potentially shallower and colder mantle melting in the western segment where Methana is located, and deeper and hotter melting that can occur in the southern segment of the volcanic arc. The temperature ranges explored in plot (**B**) illustrate the sensitivity of the dissolved water content of the primary melts to even small changes in the temperature of partial melting.

The reason why the volcanic activity ceases after the mantle source reaches its most contaminated and likely metasomatized state (*40*) could be related to the generation of superhydrous melts (>6 wt % dissolved H_2_O), which are even richer in volatiles than the damp melts responsible for the explosive events (4 to 5 wt % H_2_O). Their generation is consistent with pressure-temperature-H_2_O–dependent models of magma production (*76*, *77*), indicating that by melting mantle domains that are increasingly more hydrated, for example, by sediment-derived melts, the primary melts acquire progressively higher amounts of dissolved water content. This remains valid even at the moderate to relatively high degrees of partial melting required to produce calc-alkaline magmas, like those observed at Methana. We illustrate this effect in Fig. 8, based on the parameterization of (*77*). Similarly, for a given source hydration, melting at higher ambient pressures or lower temperatures yields higher water concentrations in primary melts, although this could increase the alkalinity in case of lower degrees of partial melting (which, in this case, would require lower levels of mantle hydration). Therefore, a variety of scenarios involving intensely hydrated mantle domains melting at higher pressures and/or lower temperatures can readily produce superhydrous primary magmas with >6 wt % dissolved H_2_O. Moreover, even damper primary melts, with 4 to 5 wt % dissolved H_2_O, can evolve into superhydrous mafic melts during lower-crustal differentiation (after ~20 to 30% crystallization), implying that such magmas may be more common in arc settings than previously thought.

The superhydrous melts, following the behavior illustrated in Fig. 7, have an increased chance of stalling at upper-crustal levels, not only due to their reduced mobility but also due to the presence of the magma reservoir which acts as an additional trap. In this case, the superhydrous melts contribute to the growth of the subvolcanic reservoir, with little to no volcanic output, generating a long period of repose.

In summary, a prolonged period of quiescence, of >100,000 years, is not necessarily a sign of volcanic death but could also indicate the growth and evolution of the magmatic system. The key to differentiating between the two scenarios is related, at least partly, to the evolution of the mantle source. If the isotopic record preceding the volcanic pause indicates a relatively depleted, refractory source (e.g., positive εHf or εNd and low ^87^Sr/^86^Sr), which, in arc settings, may be associated with lower production of melts in the mantle (*40*), then the subvolcanic magma reservoir is expected to cool and crystallize, tending toward its solidus. If the isotopic and geochemical records indicate a strong input of recycled crustal material into the mantle before the volcanic pause, the quiescence can be induced by a period of intrusive activity (decreasing the volcanic-plutonic ratio) related to the upper-crustal stalling of superhydrous melts and growth of the subvolcanic magma reservoir.

Volcanoes that undergo long dormancy periods associated with silent magma reservoir growth are still expected to remain geophysically active throughout this evolutionary stage. Sustained mafic recharge and accumulation of magma in the crust should result in volcano-tectonic earthquakes, electromagnetic, ground deformation, and gravity anomalies, these signals indicating which long-dormant volcanoes (>10 kyr) are prone to reawakening (*78*). This highlights the importance of monitoring dormant volcanoes, even in the absence of recent eruptions.

### Outlook

Petrochronological evidence from the Methana volcano (Bay of Athens, Greece) shows that magma reservoirs can undergo nucleation and growth without concurrent volcanic eruptions for long periods of time. This silent reservoir growth can be linked to the generation in the mantle of superhydrous primitive melts (>6 wt % dissolved H_2_O) that can effectively stall at upper-crustal levels due to en-route water saturation with related crystallization and increase in viscosity (*63*, *74*, *75*). The same mechanism can be responsible for extended periods of volcanic quiescence of over 100,000 years, such as recorded at Methana, which could deceptively lead to a volcano being classified as extinct, while it actually undergoes a period of magma reservoir growth. Such an episode can lead to a larger magmatic system, which, upon reaching sufficiently high volumes of upper-crustal storage, can become a recharge sponge. Under such conditions, the system has a lower volcanic-plutonic ratio but gains the ability to extrude higher volumes of material during less frequent eruptions. It would allow the system to depart from the classical behavior of arc volcanoes that erupt small-volume, crystal-rich intermediate magmas, where eruptions are easily triggered by recharge of relatively dry mafic magmas (*3*, *50*). This reservoir growth may explain how some magmatic systems reach sizes capable of generating large volumes of extractable rhyolitic melts, such as the Aegean counterpart of Methana, the Kos-Nisyros volcano, which has led to catastrophic caldera-forming eruptions. Reaching such a state would require either several growth episodes like the one inferred during Methana’s 110 kyr of quiescence or a longer period of reservoir growth supported by a sustained flux of superhydrous melts originating from a metasomatized mantle source.

## MATERIALS AND METHODS

### Study design

Fresh rock samples were collected from 31 of the 34 mapped units of Methana volcano, for dating and geochemical analyses that allow us to track the evolution of the volcanic system in time. Global Positioning System coordinates are available in table S1. The samples were cut into billets, and the crystals detached from the matrix glass using high-voltage selective fragmentation, a batch processing technique that exposed the sample (300 to 500 g of material) to electrical pulses of 115 to 125 kV. The material (50 to 100 g per sample) was subsequently separated into light and heavy fractions by heavy liquid separation using a sodium polytungstate solution with a density of 2.89 g/cm^3^. Zircon and plagioclase crystals, including ilmenite for the basaltic andesite sample, were handpicked under a stereographic microscope and prepared for dating and isotopic analyses by mounting them in epoxy resin, followed by grinding with aluminum oxide paper and polishing with diamond suspension. A piece from each rock sample was also cut for bulk rock x-ray fluorescence analyses. The methodological details of the analyses, which were all performed in the laboratories of ETH Zürich, Switzerland, are provided below. The data, including reference materials, are provided in the Supplementary Materials (tables S1 to S3).

### U-Th and U-Pb dating

For zircon dating, we ran a preliminary U-Th survey on six crystals from each sample, based on which we selected the preferred methods: U-Th disequilibrium dating for units generally younger than 200 ka and U-Pb dating for older units, which were then analyzed in separate sessions. For both methods, dating was performed with an LA-ICP-MS setup, consisting of a Resonetics Resolution S155 laser ablation system connected to a Thermo Element XR sector field mass spectrometer. The ablation spots were set to a diameter of 29 μm, and the laser was configured to a repetition rate of 5 Hz and a fluence of 2.5 Jcm^−2^. The ^230^Th/^232^Th, ^238^U/^232^Th, and ^238^U/^230^Th ratios and the ^207^Pb/^235^U and ^206^Pb/^238^U ratios, respectively, were measured. Ablation time was 40 s, following a gas blank acquisition of 30 to 40 s. Zircon crystals varied in size but were mostly between 50 and 200 μm. Smaller grains typically accommodated only a single U-Th or U-Pb ablation spot, while larger crystals allowed us to target both rims and cores, to capture both younger and older crystallization domains.

For U-Th disequilibrium dating, gas blank–corrected intensities and uncertainties on the ratios were obtained using the SILLS software (*79*). The complete data reduction followed the methodology described in (*28*) and consists of correcting for interferences on ^230^Th caused by ablation-induced polyatomic zirconium oxides, for the abundance sensitivity of ^232^Th on ^230^Th using monazite measurements, and for the relative sensitivity factor based on zircon 91500 (*80*) and NIST 612 glass. Secular equilibrium zircon reference materials, including Plesovice (*81*), Fish Canyon Tuff (*82*), GJ-1 (*83*), and AUSZ7-1 (*84*), were measured repeatedly throughout the sessions to show the accuracy of the corrections (table S2).

To calculate individual crystallization ages based on U-Th isotopic data, we relied on the two-point isochron method, where isochrons are constructed for each individual zircon based on the equilibrium melt, which, in this case, was assumed to be the groundmass glass of each unit (*6*, *85*). The isotopic composition of the groundmass glasses were analyzed with the same setup as above but with laser spots of 163 μm, a repetition rate of 10 Hz, and a fluence of 3.5 Jcm^−2^. Data reduction was performed similarly to zircon but without the zirconium polyatomic oxide correction which is not necessary in this case. The reference materials used are ATHO-G (*86*), BCR-2G (*87*), BHVO-2G (*87*), and T1-G (*86*).

For the U-Th disequilibrium dating of ilmenite crystals, we used the method described in (*29*). The laser spots were varied according to crystal size between 100 and 257 μm, the repetition rate was 20 Hz, and the ablation duration was 30 s, with 40 s of gas blank measurement. The same corrections as for the isotopic analyses of groundmass glass were performed, using ilmenites in secular equilibrium from the Fish Canyon Tuff, as well as rutile and titanite in secular equilibrium, and glass (GSD-1G and GSE-1G) (*87*) for the relative sensitivity factor correction and as validation reference material. Data are available in table S2.

For U-Pb dating, the data reduction was done with the Iolite software (*88*) using the U-Pb Geochronology reduction scheme with VisualAge (*89*), which includes a downhole fractionation routine and GJ-1 (*83*) as the primary reference material. Additional corrections, namely, for common lead and initial Th disequilibrium, were performed using IsoplotR (*90*), which also yielded the corrected crystallization ages. For the Th disequilibrium correction, the initial isotopic activity ratio is equivalent to the Th/U fractionation factor between the crystal and the melt, and we used a nominal value of 0.2 (*91*). Some points were not concordant on the Tera-Wasserburg plot due to small amounts of common Pb, and we based the common lead correction on the isochron intercept whenever the ratios defined clear isochrons in the concordia space. Otherwise, we used the Stacey-Kramers model (*92*) to determine the ^207^Pb/^206^Pb (0.8356) of the present day. The secondary standards that were run throughout the measurement sessions include AUSZ8-10 (*93*), AUSZ7-5 (*94*), AUSZ7-1 (*84*), Penglai (*95*), 91500 (*80*), Plesovice (*81*), and Temora (*96*). Data are available in table S2.

### Hf and Sr isotopes

Hafnium isotope compositions of zircon (table S3) were determined by LA-MC-ICP-MS, coupling an Australian Scientific Instruments RESOlution 193-nm ArF excimer laser to a Nu Instruments Plasma 2 multicollector inductively coupled plasma mass spectrometer. The measurements were carried out with a spot size of 50 μm over 40 s, an energy fluence of 4 Jcm^−2^, and a repetition rate of 5 Hz. To correlate age and Hf isotope data, we placed the Hf spot directly overlapping the initial U-Th or U-Pb crater, which expanded the diameter of the original pit from 29 to 50 μm, at a similar ablation depth of ~15 μm. Because the Hf analysis does not preferentially ablate at the base of the original crater, significant mass fractionation effects are not expected. Mud Tank zircon was used as the primary reference material, and raw data were reduced using Iolite 4 and the Hf isotopes data reduction scheme. The radiogenic ^176^Hf/^177^Hf isotopic ratio was corrected for mass bias and interferences of ^176^Yb and ^176^Lu, determined using the mass bias–corrected ratios ^176^Yb/^173^Yb = 0.79502 and ^176^Lu/^175^Lu = 0.02656. Initial Hf isotopic composition was calculated using the measured ^176^Hf/^177^Hf and ^176^Lu/^177^Hf ratios, the crystallization age obtained for the corresponding ablation spot, and the decay constant of (*97*) for ^176^Lu (1.867·10^−11^). Uncertainty on the Hf isotopic composition is reported as 2 SE and is calculated by quadratic addition of within-run 2 SE analytical uncertainty with the average 2 SD reproducibility on the initial ^176^Hf/^177^Hf ratios of zircon reference materials. εHf (t) was calculated using the chondrite uniform reservoir parameters (*31*). GJ-1 (*98*), Temora (*99*), and Plesovice (*81*) validation reference materials were analyzed together with the sample unknowns to test the method accuracy. Analyses with a high Yb/Hf ratio (^176^Yb/^177^Hf > 0.1) were discarded.

Strontium isotope compositions of plagioclase were determined using the same LA-MC-ICP-MS setup as above, following the procedure outlined in (*100*). Plagioclase was analyzed with a repetition rate of 8 to 10 Hz, an energy density of 3.5 to 4.0 Jcm^−2^, and a spot size of 100 μm for the unknowns and for most standards, including Hrappsey 14-2, but smaller for the plagioclase standards AMNH-107160 and G29958 (*101*). Plagioclase standards and unknowns were ablated for 40 s, preceded by 30 s of gas blank measurements, and followed by 30 s of sample washout. The following masses were monitored: 88, 87, 86.5, 86, 85.5, 85, 84.5, 84, 83.5, 83, and 82. Krypton corrections on masses 84 and 86 were performed by on-peak baseline correction. The mass bias was corrected using an exponential law and a reference ^86^Sr/^88^Sr ratio of 0.1194. The intensity of ^85^Rb and a fixed ^87^Rb /^85^Rb = 38.562 were used to correct the ^87^Rb interference on ^87^Sr. Ca dimer and argide interferences were monitored at masses 82 and 83 and corrected accordingly. No correction for isobaric interference of doubly charged REE was required due to negligible REE contributions, never exceeding background values. The data were reduced using the Iolite 4 software (*88*).

Total Sr signals varied between ~1.1 and 2.7 V for plagioclase unknowns. Analytical accuracy and instrumental drift were evaluated by repeated ablation (every ~15 measurements of plagioclase unknowns) of isotopically homogeneous plagioclase standards with G29958 used as the primary reference material. All data are reported relative to G29958 ^87^Sr/^86^Sr of 0.707551 (*101*) via standard bracketing. The weighted mean ^87^Sr/^86^Sr for AMNH-107160 and Hrappsey 14-2 is consistent with solution analyses of the same material reported in (*101*) (table S3). No correction for ^87^Sr ingrowth was applied due to the very young ages (<1 million years) of the examined samples coupled with low ^87^Rb/^86^Sr (generally < 0.05). Measurements of ^84^Sr/^86^Sr for unknowns and standards are consistent with the accepted value of ~0.0565. Data screening was based on total signal (>1 V), ^84^Sr/^86^Sr and Rb/Sr. Anomalous parts (e.g., high Rb/Sr) of the spectra were removed from integration.

### Bulk rock chemical analyses

For determining bulk rock compositions, unaltered rocks (lavas and pumices) were powdered, dehumidified at 110°C for 24 hours, devolatilized at 900°C for 2 hours, and fused into glass discs at 1200°C, after mixing with a lithium tetraborate flux. The bulk rock analyses were performed with a PANalytical AXIOS wavelength dispersive x-ray fluorescence spectrometer. The trace element analyses were done on the same fused glasses, with a laser ablation system consisting of a 193-nm ArF-Excimer (Geolas) laser connected to an NexION 2000 ICP mass spectrometer. The glasses were ablated over 100 μm for 40 s at a repetition rate of 10 Hz. Each sample was measured three times, and the results were averaged (table S1).
