# Supplementary material for: A volcano reawakens after more than 100,000 years of “silent” magma reservoir growth
Source: Sci Adv. 2026 Apr 22;12(17):eaec9565. doi: 10.1126/sciadv.aec9565 (PMC13101864; doi:10.1126/sciadv.aec9565)
Supplement: Supplementary file 1 — Legends for tables S1 to S3 [file sciadv.aec9565_sm.pdf]

Science Advances
AAAS

Supplementary Materials for

A volcano reawakens after more than 100,000 years of “silent” magma reservoir growth

Răzvan-Gabriel Popa *et al.*

Corresponding author: Răzvan-Gabriel Popa, razvan.popa@eaps.ethz.ch

Sci. Adv. **12**, eaec9565 (2026)
DOI: 10.1126/sciadv.aec9565

The PDF file includes:

Legends for tables S1 to S3

Other Supplementary Material for this manuscript includes the following:

Tables S1 to S3

Table S1. (separate file)

Methana volcano: dating and compositional overview. The table reports GPS coordinates of sampling sites, eruption ages, and major oxide (X-ray Fluorescence) and trace element (LA-ICP-MS) bulk-rock compositions. Uncertainties, limits of detection, and secondary reference material analyses are provided for the compositional data.

Table S2. (separate file)

U-Th-Pb isotopic data in zircon, ilmenite, and groundmass glass. *In situ* LA-ICP-MS analyses (Laser Ablation Inductively Coupled Plasma Mass Spectrometry) are reported together with calculated crystallization ages and crystallization-age rank plots for individual eruptions. Secondary reference material data are also included.

Table S3. (separate file)

Lu-Hf isotopic data in zircon and Sr isotopic data in plagioclase. *In situ* MC-ICP-MS (Multi-Collector Inductively Coupled Plasma Mass Spectrometry) analyses are presented along with the secondary reference materials.
